# Bayesian network analysis of panomic biological big data identifies the importance of triglyceride-rich LDL in atherosclerosis development

**DOI:** 10.3389/fcvm.2022.960419

**Published:** 2023-01-04

**Authors:** Szilard Voros, Aruna T. Bansal, Michael R. Barnes, Jagat Narula, Pal Maurovich-Horvat, Gustavo Vazquez, Idean B. Marvasty, Bradley O. Brown, Isaac D. Voros, William Harris, Viktor Voros, Thomas Dayspring, David Neff, Alex Greenfield, Leon Furchtgott, Bruce Church, Karl Runge, Iya Khalil, Boris Hayete, Diego Lucero, Alan T. Remaley, Roger S. Newton

**Affiliations:** ^1^Global Genomics Group, Atlanta, GA, United States; ^2^Acclarogen, Ltd, Cambridge, United Kingdom; ^3^Mount Sinai School of Medicine, New York, NY, United States; ^4^MTA-SE Cardiovascular Imaging Research Group, Heart and Vascular Center, Semmelweis University, Budapest, Hungary; ^5^Global Institute for Research, LLC, Richmond, VA, United States; ^6^OmegaQuant, Sioux Falls, SD, United States; ^7^Department of Psychiatry, Medical School, University of Pécs, Pécs, Hungary; ^8^GNS Healthcare, Somerville, MA, United States; ^9^Lipoprotein Metabolism Laboratory, National Heart, Lung and Blood Institute, National Institutes of Health, Bethesda, MD, United States

**Keywords:** triglyceride-rich LDL, LDL-triglycerides, cardiovascular risk, Bayesian network analysis, omics, hepatic lipase

## Abstract

**Introduction:**

We sought to explore biomarkers of coronary atherosclerosis in an unbiased fashion.

**Methods:**

We analyzed 665 patients (mean ± SD age, 56 ± 11 years; 47% male) from the GLOBAL clinical study (NCT01738828). Cases were defined by the presence of any discernable atherosclerotic plaque based on comprehensive cardiac computed tomography (CT). De novo Bayesian networks built out of 37,000 molecular measurements and 99 conventional biomarkers per patient examined the potential causality of specific biomarkers.

**Results:**

Most highly ranked biomarkers by gradient boosting were interleukin-6, symmetric dimethylarginine, LDL-triglycerides [LDL-TG], apolipoprotein B48, palmitoleic acid, small dense LDL, alkaline phosphatase, and asymmetric dimethylarginine. In Bayesian analysis, LDL-TG was directly linked to atherosclerosis in over 95% of the ensembles. Genetic variants in the genomic region encoding hepatic lipase (LIPC) were associated with LIPC gene expression, LDL-TG levels and with atherosclerosis.

**Discussion:**

Triglyceride-rich LDL particles, which can now be routinely measured with a direct homogenous assay, may play an important role in atherosclerosis development.

**Clinical trial registration:**

GLOBAL clinical study (Genetic Loci and the Burden of Atherosclerotic Lesions); [https://clinicaltrials.gov/ct2/show/NCT01738828?term=NCT01738828&rank=1], identifier [NCT01738828].

## Highlights

- In our Bayesian analysis, LDL-TG was directly upstream from atherosclerosis.
- LDL-TG was associated with atherosclerosis independently of well-known factors.
- Hepatic lipase’s genetic variants correlated with LDL-TG levels and atherosclerosis
- LDL-TG was positively linked to triglycerides, sd-LDL, and inflammatory markers.

## Introduction

Cardiovascular disease remains the leading cause of mortality and morbidity worldwide (1). Acute manifestations of coronary artery disease (CAD) are caused by at least three relevant biological processes: underlying coronary arterial atherosclerosis that develops over decades (2), acute plaque rupture/erosion (3) followed by coronary arterial thrombosis (4). Many long-term studies of cardiovascular outcomes have identified low-density lipoprotein cholesterol (LDL-C) and apolipoprotein-B (Apo-B) as key causal risk factors for cardiovascular events (5, 6).

Such long-term cardiovascular outcomes studies are very helpful in establishing clinically relevant risk factors that can be monitored and modified in clinical practice, such as LDL-C and Apo-B. A limitation of the current cardiovascular biomarker studies is that they primarily rely on clinical events, which is a combination of the three underlying biological processes with different time scales, namely atherogenesis, plaque rupture/erosion and thrombosis. As a consequence, the current cardiovascular biomarker studies do not efficiently identify and discriminate which of these three specific biological processes is associated with and causally linked to a risk factor.

Non-invasive coronary arterial imaging with cardiac CT presents a unique opportunity to isolate causal factors of atherosclerosis *per se*. Accordingly, we designed a nested case-control analysis within the Genetic Loci and the Burden of Atherosclerotic Lesions (GLOBAL) clinical study (ClinicalTrials.gov number NCT01738828) (7) to identify additional causal factors. We used *de novo* Bayesian network analysis, a hypothesis-free approach (8), to enrich for associations with risk of CT for causal relevance to the development of atherosclerosis. In order to examine causal relevance of relationships among the multi-modal covariates of CAD (genetics, gene expression, proteomics, etc.), we used a specific technique successfully employed to infer biological pathways from steady-state cross-sectional data, namely Bayesian belief networks (8, 9). In addition, our network analysis incorporated whole genome sequencing data and other data modalities to avoid latent confounding and to study potential causal biomarkers revealed by Bayesian network analysis.

## Materials and methods

### Patients

The analyses for the present study were performed in a subgroup from the Genetic Loci and the Burden of Atherosclerotic Lesions (GLOBAL) multicentric clinical study (ClinicalTrials.gov number NCT01738828). The present nested Case-Control study was performed in the pre-specified Pilot Discovery (340 patients) and Pilot Validation (340 patients) cohorts, which, in combination, included 680 patients. Entirely complete clinical, imaging, multiomic and genetic data with zero missingness that is required to build the integrated data frame for Bayesian analysis was available in 665 patients. Of these, 317 subjects had no discernable atherosclerosis on comprehensive CT and were therefore designated as “Controls” and 348 subjects had discernable plaque on CT and were designated as “Cases.” The GLOBAL study included subjects of 18–90 years of age and self-referred as Caucasian, with the indication of or undergoing coronary computerized tomography (CT). Subjects under immunosuppressive or immunomodulatory therapy or chemotherapy were excluded from the study. Those with major surgery and blood transfusion within the last two months, contraindicated CT, or preexisting cardiac affections were also excluded from the study. Blood draw for all blood-based biomarker analysis, “omics” testing and genetic testing was performed at the time of the CT imaging procedure. For further details about the GLOBAL study design, please go to Voros et al. (7). The study was conducted according to the criteria set by the declaration of Helsinki and all included subjects signed informed consent for the use of genetic material for research purposes. The study was approved by institutional review boards and ethics committee as appropriate. Cardiac CT was evaluated as previously described (7). Subjects with any evidence of atherosclerotic plaque in coronary CT were considered cases, and those without, controls. Peripheral blood samples were obtained from enrolled subjects, and plasma, serum, whole blood, and buffy coat were adequately stored for further analysis.

### Data analysis approach

In order to examine causal relevance of relationships among the multi-modal covariates of CAD (genetics, gene expression, proteomics, etc.), we used Bayesian belief networks (8, 9). Although Bayesian Belief networks may not always be able to establish a unique relationship between covariates, we relied on Markov equivalence to take advantage of additional information in order to break synonymous probabilistic relationships. This approach can take the form of intentional perturbations (8) or of genetic constraints, the use of which in Bayesian networks permits analysis that is statistically related to mendelian randomization (10). Furthermore, we investigated our networks of causally enriched probabilistic relationships by means of per-patient counterfactual simulations (11), where the genetic constraints played a role similar to that of instruments in instrumental variable analysis. In addition, our networks incorporated our whole genome sequencing data and other data modalities to avoid latent confounding and to study potential causal biomarkers revealed by Bayesian network analysis.

### Detailed sample size calculations for Bayesian network analysis

**FIGURE 1:**
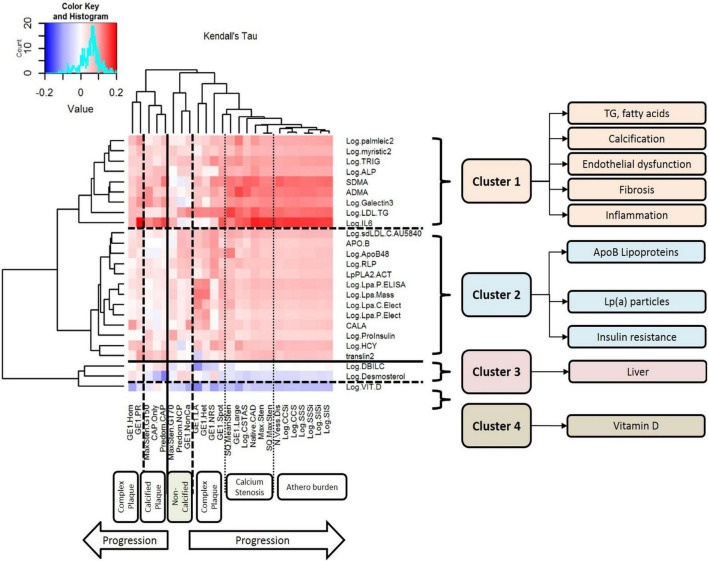
Clusters of circulating biomarkers against CT measures of atherosclerotic plaque, stenosis, and disease burden. This figure displays the results of unsupervised hierarchical clustering of circulating biomarkers against CT measures of atherosclerotic plaque, stenosis, and disease burden. Each row represents a biomarker that was nominally associated with atherosclerosis, and each column represents a CT measurement of atherosclerosis. The dendrograms on both axes show the results of hierarchical cluster analysis. Inside the heat map, positive correlations are shown in red and negative correlations are shown in blue; the intensity of the color represents the strength of association, as quantified by Kendall’s tau. There are six major clusters of CT measurements of atherosclerosis: complex plaque, calcified plaque, non-calcified plaque, calcification/stenosis, disease burden, and a second cluster of complex plaque. There are four clusters of circulating biomarkers with different patterns of association with different measures of atherosclerosis: Cluster 1 includes triglycerides, fatty acids, calcification, endothelial dysfunction, fibrosis, and inflammation. LDL-TG is strongly associated with early-stage, non-calcified plaque and complex plaque, and IL-6 is strongly associated with later-stage, calcified plaque and atherosclerosis burden. Cluster 2 includes ApoB-containing lipoproteins, lipoprotein (a), and biomarkers of insulin resistance; Cluster 3 includes hepatic biomarkers and markers of hepatic cholesterol synthesis; and Cluster 4 contains vitamin D alone.

Tanner and Donoho have pioneered a compressed sensing approach which bounds inferable complexity given available data and assumed sparseness (12). In their simulations, for example, if number of samples, *n* = 300, number of useful predictors, *k*, is 3 on average, and number of variables, *p*, is 100,000 (as in our case), the x-axis – delta – in Figure 1 is *n*/*p* = 300/100000 ∼ 0, the worst possible case, but the y-axis – rho – is *k*/*n* = 3/300 ∼ 0 << 0.15. While the specific numbers do not map to our problem domain, they illustrate that statistical inference depends on *k*, *n*, and *p*, and in our case is expected to be very hard. In order to ensure that we do not suffer from overfitting, we have applied a number of priors, as documented in the manuscript. In particular, these include the probability of the local model (modeled by BIC, or penalized likelihood) multiplied by the prior probability of the model of a given complexity and has been described by us in the supplement to a prior work (13). In the case of a single class (e.g., only gene expression), the total overall penalty simplifies to E-BIC with gamma = 1/2, or simply BIC + log(| S|), where | S| is the number of all possible models (network fragments) of the same size as S. When multiple data types are present, our incremental penalty for adding a term of class C to a model is defined as deltaBIC + log(| C|) + log(| S_c|), where deltaBIC is the change in BIC due to this addition, | C| is the number of classes, and | S_c| is the number of elements in class C. Effectively, this formula computes E-BIC subject to the Bayesian belief that all classes are equally informative *a priori*, before any data is seen, thus penalizing large classes, e.g., genetics, more than small ones, e.g., clinical data. Subject to this regularization strategy, the network’s default state is to be fully disconnected, and it can only become connected through the preponderance of evidence that overcomes these two penalties. Further, the use of large model ensembles makes it virtually impossible that the network overtrains systematically; in a way that would be repeatable in simulations. Any overtraining would be diluted by the entropy of the ensemble. Performing simulations on a per-network basis and averaging their predictions allows us to shrink the overall standard error of the estimate by the aforementioned dilution of errors. This property of ensemble methods is well-studied and has been reflected in a number of popular approaches to classification and regression, as described in the manuscripts cited above.

#### Conventional biomarker analysis

A panel of conventional biomarkers were evaluated using commercially available kits and reagents, as listed in Supplementary Table 2.

### Isolation of genomic deoxyribonucleic acid

Isolation of genomic DNA was performed using the QIAamp DNA Blood Midi Kit (Qiagen part no. 51185). Starting with 0.3–1 ml of whole blood, a lysis buffer and protease were added to each sample for cell lysis. After lysis, the lysate was loaded onto a QIAamp spin column. DNA remained bound to the QIAamp membrane, while impurities were washed away in 2 vacuum steps. Upon drying the membrane, DNA was eluted in 200 μl of elution buffer. The yield of genomic DNA was subsequently determined by PicoGreen quantitation or by using the Qubit fluorometer.

### Whole genome sequencing (Illumina Service Laboratory)

Whole-genome sequencing was performed by the Illumina Service Laboratory.

#### Genomic deoxyribonucleic acid quantitation

Genomic DNA was quantified prior to library construction using PicoGreen (Quant-iT™ PicoGreen^®^ dsDNA Reagent, Invitrogen, Catalog #: P11496). Quants were read with Spectromax Gemini XPS (Molecular Devices).

#### Library construction – Polymerase chain reaction-free

Paired-end libraries were manually generated from 500 ng to 1 μg of genomic DNA using the Illumina TruSeq DNA Sample Preparation Kit (Catalog #: FC-121-2001), based on the protocol in the TruSeq DNA PCR-free Sample Preparation Guide. Prefragmentation genomic DNA cleanup was performed using paramagnetic sample purification beads (Agencourt^®^ AMPure^®^ XP reagents, Beckman Coulter). Samples were fragmented and libraries were size-selected following fragmentation and end-repair using paramagnetic sample purification beads, targeting 300 bp inserts. Final libraries were quality controlled for size using a gel electrophoretic separation system and were quantified.

#### Clustering and sequencing – v3 chemistry

Following library quantitation, DNA libraries were denatured, diluted, and clustered onto v3 flow cells using the Illumina cBot™ system. cBot runs were performed based on the cBot User Guide, using the reagents provided in Illumina TruSeq Cluster Kit v3. Clustered v3 flow cells were loaded onto HiSeq 2000 instruments and sequenced on 100 bp paired-end, non-indexed runs. All samples were sequenced on independent lanes. Sequencing runs were performed based on the HiSeq 2000 User Guide, using Illumina TruSeq SBS v3 Reagents. Illumina HiSeq Control Software and Real-time Analysis were used on HiSeq 2000 sequencing runs for real-time image analysis and base calling.

#### Genotyping

Samples were processed using Infinium chemistry, based on the Infinium LCG Assay Guide, and run on the HumanOmni2.5-8 array. Resulting intensity.idat files were loaded into GenomeStudio^®^ software to export genotyping calls.

### Ribonucleic acid isolation from PAXGene tubes

RNA isolation was completed using the PAXgene Blood miRNA Kit (Qiagen, Venlo, The Netherlands). PAXgene Blood RNA Tubes were first centrifuged to pellet the samples, then washed with water and resuspended. After digestion with proteinase K, the samples were homogenized by centrifugation through PAXgene Shredder spin columns. Isopropanol was added to the samples to optimize binding conditions, and the samples were then centrifuged through PAXgene RNA spin columns, where total RNA >18 nucleotides (including miRNA) was bound to the silica membrane. The bound RNA was treated with DNase to remove genomic DNA contamination and washed. Pure RNA was then eluted.

### Small ribonucleic acid sequencing methods and materials

Libraries were prepared for small RNA sequencing using the TruSeq Small RNA Sample Prep Kit (Illumina). Prior to library preparation, RNA samples were quantitated by spectrophotometry using a Nanodrop ND-8000 spectrophotometer and assessed for RNA integrity using an Agilent 2100 BioAnalyzer or Caliper LabChip GX. RNA samples with A260/A280 ratios ranging from 1.6 to 2.2, with RNA integrity number values ≥7.0, and for which at least 1,000 ng of total RNA was available proceeded to library preparation. Total RNA samples must have been prepared using extraction chemistry that does not exclude small RNA species (e.g., the QIAGEN miRNeasy Kit).

Library preparation began with 1,000 ng of total RNA in 5 μl of nuclease-free water, to which an adapter oligonucleotide was added that was then ligated to the 3′ hydroxyl present on miRNA species using T4 RNA ligase (New England Biolabs). Similarly, a different adapter sequence was ligated to the 5′ end of RNAs that possessed a 5′ phosphate, in order to create a single-stranded molecule with defined sequences at both the 5′ and 3′ ends. This molecule was reverse-transcribed and amplified using 14 cycles of PCR with primers that include sequences complementary to the 5′ and 3′ adapter sequences, a specific index sequence, and Illumina sequencing adapter sequences. The resulting product was analyzed using an Agilent 2100 BioAnalyzer, and the molar amount of mature miRNA present in the library was estimated by integrating the area under the curve in the 145–160 bp range. Individual libraries were mixed to create multiplexed pools, and the mixture was purified by gel electrophoresis, wherein the 145–160 bp range was excised from the gel, crushed using a Gel Breaker tube (IST Engineering), eluted into nuclease-free water, and concentrated by precipitation with ethanol. The concentration of the final library pool was determined using PicoGreen (Invitrogen), and the size distribution of the pool was determined using an Agilent 2100 BioAnalyzer. Library pools were normalized to 2 nM in preparation for sequencing.

### mRNA sequencing

Prior to library preparation, alpha and beta globin mRNA was reduced using the GLOBINclear™-Human Kit (Life Technologies, Carlsbad, CA), following the manufacturers protocol. Total RNA samples were converted into cDNA libraries using the TruSeq Stranded mRNA Sample Prep Kit (Illumina, #RS-122-2103). Starting with 100 ng of total RNA, polyadenylated RNA (primarily mRNA) was selected and purified using oligo-dT conjugated magnetic beads. This mRNA was chemically fragmented and converted into single-stranded cDNA using reverse transcriptase and random hexamer primers, with the addition of Actinomycin D to suppress DNA-dependent synthesis of the second strand. Double-stranded cDNA was created by removing the RNA template and synthesizing the second strand in the presence of dUTP instead of dTTP. A single A base was added to the 3′ end to facilitate ligation of sequencing adapters, which contained a single T base overhang. Adapter-ligated cDNA was amplified by polymerase chain reaction to increase the amount of sequence-ready library. During this amplification, the polymerase stalls when it encounters a U base, rendering the second strand a poor template. Accordingly, amplified material used the first strand as a template, thereby preserving the strand information. Final cDNA libraries were analyzed for size distribution using an Agilent BioAnalyzer (DNA 1000 Kit, Agilent #5067-1504), quantitated by qPCR (KAPA Library Quant Kit, KAPA Biosystems #KK4824), and then normalized to 2 nM in preparation for sequencing.

### Mass-spectrometry–based proteomics methods

We performed proteomics discovery experiments in 2 stages; the first stage was performed using non-targeted mass spectrometry, followed by the second stage of targeted mass spectrometry using multiple reaction monitoring.

### Discovery experiments using non-targeted mass spectrometry

Samples were processed essentially as described previously (14). Briefly, each 30 μl sample was depleted of high abundance proteins using an affinity resin (IgY14/Supermix, Sigma). All columns were prepared with the same manufacturing batch of affinity resin and tested for consistent performance prior to use. Control samples, consisting of aliquots of a pooled human plasma sample, were inserted at the start, middle, and end of each set of 20 paired study samples, resulting in a batch size of 23. After depletion, samples were frozen, freeze-dried, digested with trypsin (1:10, w:w, Promega), and desalted on Empore C18 plates (3M Bioanalytical Technologies). Resulting peptides were separated by strong cation exchange (SCX, Waters) chromatography into 6 fractions with a linear salt gradient and desalted on Oasis HLB plates (Waters). Samples were distributed into two 96-well plates (one test plate and one backup plate). Samples were then dried and resuspended in 96.25/3.75 (v/v) water/acetonitrile and 0.1% formic acid, containing 19 internal standard peptides. Mass spectrometry analysis was performed by nanoflow reversed phase liquid chromatography (NanoAcquity UPLC, Waters), coupled by electrospray (Michrom ADVANCE CaptiveSpray MS Source) to a high-resolution mass spectrometer (Q Exactive, ThermoScientific) in liquid chromatography mass spectrometry (LC-MS) and liquid chromatography/tandem mass spectrometry (LC-MS/MS) mode. The LC column was used at a flow rate of 1.8 μl/min (Waters nanoAcquity UPLC column BEH130 C18, 150 μm × 100 mm, 1.7 μm). Each of the 6 fractions was run as a separate set of 338 samples plus control samples.

Intensity data files for each LC-MS run within a SCX fraction were aligned using Elucidator (Rosetta Biosoftware). Peak intensities for each peptide ion were then extracted across all files. LC-MS/MS files were analyzed by Mascot (Matrix Sciences) and the Uniprot human protein database (version 2013_08) to assign high confidence peptide sequences to the observed peptide ions. All sequenced peptides were then clustered by their parent proteins. Potential intensity bias introduced by sample processing and/or loss of sensitivity of the mass spectrometer over the time of the experiment was corrected by normalization. The normalization procedure was based on a regression model, which predicted log-intensity level on a per-peptide basis. First, the mean raw log-intensity for each peptide was calculated. Then the regression model (linear regression or natural cubic spline smoothing) for sample processing variables was fit to the data. Finally, the normalized log-intensity was computed as the raw log-intensity minus the regression-predicted log-intensity plus the mean raw log-intensity.

The statistical significance of the intensity differences between the various clinical groups was assessed using a paired *t* test, which was performed independently on each peptide and each protein, for the matched case and control samples. An analysis of variance model was also used to compare the same two groups to account for dyslipidemia, hypertension, and diabetes status covariates, which were not matched between sample pairs. All statistical test *P* values were adjusted for multiple testing by conversion to *Q* values using Storey’s method.

### Metabolomics and lipidomics methods by mass spectrometry

#### Sample preparation for global metabolomics

Samples were stored at –70°C until processed. Sample preparation was carried out as described previously (15) at Metabolon, Inc. Briefly, recovery standards were added prior to the first step in the extraction process for quality control purposes. To remove protein, dissociate small molecules bound to protein or trapped in the precipitated protein matrix, and to recover chemically diverse metabolites, proteins were precipitated with methanol under vigorous shaking for 2 min (Glen Mills Genogrinder 2000), followed by centrifugation. The resulting extract was divided into 4 fractions: 1 for analysis by ultra-high performance liquid chromatography tandem mass spectrometry (UPLC-MS/MS; positive mode), 1 for analysis by UPLC-MS/MS (negative mode), 1 for analysis by gas chromatography–mass spectrometry (GC-MS), and 1 sample was reserved for backup.

Three types of controls were analyzed in concert with the experimental samples: samples generated from a pool of human plasma (extensively characterized by Metabolon, Inc.) served as technical replicates throughout the data set; extracted water samples served as process blanks; and a cocktail of standards spiked into every analyzed sample allowed for instrument performance monitoring. Instrument variability was determined by calculating the median relative standard deviation (RSD) for the standards that were added to each sample prior to injection into the mass spectrometers (median RSD = 5%; *n* = 30 standards). Overall process variability was determined by calculating the median RSD for all endogenous metabolites (i.e., non-instrument standards) present in 100% of the pooled human plasma samples (median RSD = 11%; *n* = 610 metabolites). Experimental samples and controls were randomized across the platform run.

#### Mass spectrometry analysis

Non-targeted MS analysis was performed at Metabolon, Inc. Extracts were subjected to either GC-MS (16) or UPLC-MS/MS (15). The chromatography was standardized and, once the method was validated, no further changes were made. As part of Metabolon’s general practice, all columns were purchased from a single manufacturer’s lot at the outset of the experiments. All solvents were similarly purchased in bulk from a single manufacturer’s lot in sufficient quantity to complete all related experiments. For each sample, vacuum-dried samples were dissolved in injection solvent containing 8 or more injection standards at fixed concentrations, depending on the platform. The internal standards were used to assure both injection and chromatographic consistency. Instruments were tuned and calibrated for mass resolution and mass accuracy daily.

The UPLC-MS/MS platform utilized a Waters Acquity UPLC with Waters UPLC BEH C18-2.1 × 100 mm, 1.7 μm columns and a Thermo Scientific Q-Exactive high resolution/accurate mass spectrometer interfaced with a heated electrospray ionization source and Orbitrap mass analyzer operated at 35,000 mass resolution. The sample extract was dried and then reconstituted in acidic or basic LC-compatible solvents, each of which contained 8 or more injection standards at fixed concentrations to ensure injection and chromatographic consistency. One aliquot was analyzed using acidic, positive ion–optimized conditions, and the other using basic, negative ion–optimized conditions in 2 independent injections using separate dedicated columns. Extracts reconstituted in acidic conditions were gradient eluted using water and methanol containing 0.1% formic acid, while the basic extracts, which also used water/methanol, contained 6.5 mM ammonium bicarbonate. The MS analysis alternated between MS and data-dependent MS^2^ scans using dynamic exclusion, and the scan range was from 80 to 1,000 *m/z*.

The samples destined for analysis by GC-MS were dried under vacuum desiccation for a minimum of 18 h prior to being derivatized under dried nitrogen using bistrimethyl-silyltrifluoroacetamide. Derivatized samples were separated on a 5% phenyldimethyl silicone column with helium as carrier gas and a temperature ramp from 60 to 340°C within a 17-min period. All samples were analyzed on a Thermo-Finnigan Trace DSQ MS operated at unit mass resolving power with electron impact ionization and a 50–750 atomic mass unit scan range.

#### Compound identification, quantification, and data curation

Metabolites were identified by automated comparison of the ion features in the experimental samples to a reference library of chemical standard entries that included retention time, molecular weight (*m/z*), preferred adducts, and in-source fragments as well as associated MS spectra and curated by visual inspection for quality control using software developed at Metabolon. Identification of known chemical entities was based on comparison to metabolomic library entries of purified standards. Over 2,500 commercially available purified standard compounds have been acquired and registered into the Laboratory Information Management System for distribution to both the LC-MS and GC-MS platforms for determination of their detectable characteristics. An additional 250 mass spectral entries have been created for structurally unnamed biochemicals, which have been identified by virtue of their recurrent nature (both chromatographic and mass spectral). These compounds have the potential to be identified by future acquisition of a matching purified standard or by classical structural analysis. Peaks were quantified using area-under-the-curve. Raw area counts for each metabolite in each sample were normalized to correct for variation resulting from instrument inter-day tuning differences by the median value for each run-day; therefore, the medians were set to 1.0 for each run. This preserved variation between samples but allowed metabolites of widely different raw peak areas to be compared on a similar graphical scale. Missing values were imputed with the observed minimum after normalization.

#### TrueMass^®^ lipomic panel

Lipids were extracted in the presence of authentic internal standards by the method of Folch et al. (17) using chloroform:methanol (2:1 v/v). For the separation of neutral lipid classes [FFA, TAG, DAG, CE], a solvent system consisting of petroleum ether/diethyl ether/acetic acid (80:20:1) was employed. Individual phospholipid classes within each extract [PC, PE] were separated using the Agilent Technologies 1100 Series LC. Each lipid class was transesterified in 1% sulfuric acid in methanol in a sealed vial under a nitrogen atmosphere at 100°C for 45 min. The resulting fatty acid methyl esters were extracted from the mixture with hexane containing 0.05% butylated hydroxytoluene and prepared for GC by sealing the hexane extracts under nitrogen. Fatty acid methyl esters were separated and quantified by capillary GC (Agilent Technologies 6890 Series GC) equipped with a 30 m DB 88 capillary column (Agilent Technologies) and a flame ionization detector.

### Evaluation of associations between low density lipoprotein triglycerides and plasma lipoproteins

For a confirmatory study, eight hundred and six subjects were included from the National Institutes of Health CT study. The cohort included both males and females that were at least 18 years of age and with clinical indication for a coronary CT angiography. There were no additional inclusion criteria. Exclusion criteria were current pregnancy and severely decreased renal function (estimated glomerular filtration rate < 30 mL/min/1.73m2 body surface area). The study protocol was approved by the National Heart, Lung, and Blood Institute’s Institutional Review Board and all subjects provided informed consent at enrolment. ClinicalTrials.gov identifier: NCT01621594. Plasma LDL-TG was determined by homogeneous assay (Denka Seiken Co., Ltd., Tokyo, Japan), and subjects were divided according to LDL-TG terciles. Fasting lipid panel was determined by standard enzymatic methods on a Cobas 6000 analyzer (Roche Diagnostics, Indianapolis, IN, USA). LDL cholesterol and very low-density lipoprotein (VLDL) cholesterol were calculated using Sampson’s formula (18). Small dense LDL cholesterol was measured by a homogeneous assay (Denka Seiken Co, Ltd., Tokyo, Japan). Lipoprotein subclass profile was determined in Vantera Clinical NMR Analyzer (Labcorp, Burlington, NC, USA). The LipoProfile-3 or 4 algorithm was used to determine the particle number of lipoprotein subclasses: Number of triglyceride-rich lipoprotein particles (TRL-P) and the following subclasses: very small-, small-, medium- and large-TRL-P; LDL particle number (LDL-P), and its subclasses: small-, medium-, large-LDL-P; HDL particle number (HDL-P), as well as HDL subclasses: small-, medium-, and large-HDL-P. GlycA levels were determined in a Vantera Clinical NMR Analyzer (Labcorp, Burlington, NC, USA). Plasma high sensitivity (hs-CRP) was measured on the Cobas 6000 analyzer (Roche Diagnostics, Indianapolis, IN, USA).

## Results

### Demographic features and atherosclerosis in the patient population

A total of 665 patients were included in our analysis; general demographic features are shown in Supplementary Table 1. Typical angina (62 vs. 64%) and atypical angina (36.5 vs. 36%) were similar in cases and controls. In general, the mean ± SD age in the overall study population was 56 ± 11 years, 47% of patients were male, and the mean Diamond-Forrester score was 26% (range, 0–94%). LDL-C, high-density lipoprotein cholesterol (HDL-C), and triglycerides in cases and controls are also shown in Supplementary Table 1. The prevalence of atherosclerosis (i.e., cases) was 52% in the overall cohort. Seven percent of patients had a coronary calcium score of zero but had a non-calcified plaque. Predominantly non-calcified, partially calcified, and calcified plaques were present in 7, 36, and 57% of cases, respectively. Napkin ring sign, a high-risk feature by CT, was observed in 10% of patients. Moderate stenosis (50–69%) was the highest degree of stenosis in 7% of patients, and 16% of patients had moderate-to-severe stenosis (≥50% luminal stenosis). Mean ± SD segment involvement score and segment involvement score index were 2.2 ± 3.1 and 2.4 ± 3.3%, respectively.

### Biomarker associations with atherosclerosis

In a preliminary coarse filter of the biomarkers, nominal univariate associations (raw *P* < 0.05) with atherosclerosis were identified for 30 of the 99 conventional biomarkers; these are illustrated in a heatmap in Figure 1. The dendrogram on the left of the plot was generated by unsupervised hierarchical clustering and indicates four (4) clusters. Cluster 1 included total plasma triglycerides and LDL-TG, as well as fatty acids and measures of endothelial dysfunction, inflammation, and fibrosis. Cluster 2 included ApoB-containing lipoprotein measurements, lipoprotein(a), and measures of insulin resistance. Cluster 3 included hepatic measurements of bilirubin metabolism and a marker of cholesterol biosynthesis. Cluster 4 contained vitamin D alone.

**FIGURE 2:**
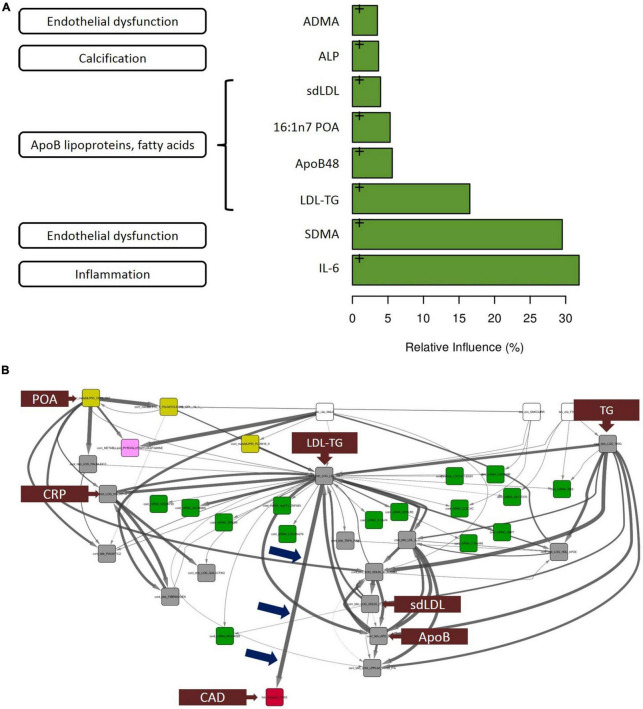
Gradient boosting analysis identified the strongest predictors of atherosclerotic coronary artery disease. Hypothesis-free Bayesian network analysis using reverse engineering with forward simulation (REFS™) Suggests a potential causal role of triglyceride-rich LDL particles, as measured by LDL-TG levels, in the development and progression of atherosclerosis. **(A)** Out of the 30 conventional biomarkers included in the multivariate analysis, the top 8 analytes are shown in the bar graph. The length of the bar corresponds to the relative influence the biomarker in predicting atherosclerotic coronary artery disease. The biomarkers include IL-6 (inflammation), symmetric dimethylarginine (endothelial dysfunction), and LDL-TG (ApoB-containing lipoprotein cluster). **(B)** This figure is not an illustration; it is an actual output from the hypothesis-free Bayesian network analysis. A total of 24,929 nodes and 110,350 edges were discovered in more than 5% of the networks in the ensemble; shown is the subnetwork of measurements with 1 degree of separation from LDL-TG. Arrow thickness indicates the fraction of networks in which the causal edge appears; different colored boxes represent different types of measurements (yellow: mass-spectrometry–based lipidomics; pink: mass-spectrometry–based metabolomics; green: gene expression [mRNA]; gray: conventional biomarker measurements). Notably, among all of the biomarkers that were measured and included in the model, LDL-TG was the only biomarker with a direct connection to ASCAD (see blue arrows pointing to the edge connecting LDL-TG to ASCAD). This suggests that triglyceride-rich lipoprotein particles, as measured by LDL-TG levels, may have a causal role in atherosclerosis. Interestingly, in this causal model, sd-LDL, ApoB, and C-reactive protein are downstream from LDL-TG, while palmitoleic acid and total triglyceride levels appear upstream from LDL-TG. Fibrinogen and galectin-3 are downstream from C-reactive protein. ADMA, asymmetric dimethylarginine; ALP, alkaline phosphatase; ApoB, apolipoprotein B; IL-6, interleukin-6; LDL-TG, low-density lipoprotein-triglycerides; SDMA, symmetric dimethylarginine, ASCAD, atherosclerotic coronary artery disease; CAD, coronary artery disease; CRP, C-reactive protein; POA, palmitoleic acid; sd-LDL, small, dense low-density lipoprotein; TG, triglycerides.

The thirty biomarkers identified by univariate analysis were further subjected to gradient boosting analysis to identify the strongest predictors of atherosclerosis Figure 2A indicates the relative influence of the eight biomarkers ranked most highly. Interleukin-6 [IL-6], symmetric dimethylarginine, and LDL-TG emerged as the top 3 predictors of case-control status, with a relative influence of over ∼30% for IL-6 and symmetric dimethylarginine and ∼15% for LDL-TG. As described below, of these eight (8) biomarkers strongly associated with atherosclerosis, only LDL-TG was directly connected to atherosclerosis in the Bayesian network analysis.

### Bayesian network analysis using reverse engineering with forward simulation

The primary result and output from the hypothesis-free Bayesian network analysis is shown in Figure 2B. The ensemble of Bayesian networks identified consisted of 24,929 nodes and 110,350 edges, which occurred in >5% of the models in the ensemble. LDL-TG was the only biomarker directly upstream from the presence of atherosclerotic CAD (ASCAD), which occurred in 95% of networks in the ensemble. This suggests a potential causal role of triglyceride-rich LDL particles, as measured by LDL-TG levels, in the development and progression of atherosclerosis. Given the central role of LDL-TG in the Bayesian networks, we further explored the potential contribution of LDL-TG to atherosclerosis. It is important to point out that clinical features, such as age and gender, were also included in the Bayesian analysis and therefore, the Bayesian findings do normalize our findings for age and gender.

### Low density lipoprotein triglycerides and atherosclerosis

As an independent biomarker, LDL-TG levels were significantly higher in cases versus controls (mean ± SE, 20.19 ± 0.93 vs 17.21 ± 0.40 mg/dL; *P* < 0.001). The four quartiles of LDL-TG measurements were examined; odds ratios in the second, third, and fourth quartiles were 1.38 (95% CI, 0.86–2.24), 1.43 (95% CI, 0.89–2.31), and 2.84 (95% CI, 1.75–4.64), respectively, compared to the first (i.e., reference) quartile (Table 1). Adjusting the model for age, sex, LDL-C and ApoB levels demonstrated that the association of LDL-TG with atherosclerosis was independent of these well-known factors.

**TABLE 1:** Odds ratios (95% CI) for atherosclerosis against the lowest quartile of LDL-TG.

	Quartile 1 [8.5–14.1 mg/dl]	Quartile 2 [14.1–16.9 mg/dl]	Quartile 3 [16.9–22.1 mg/dl]	Quartile 4 [22.1–45.7 mg/dl]	*P*-value versus fourth quartile	*P*-value for trend
LDL-TG Unadjusted	Reference	1.38 (0.86–2.24)	1.43 (0.89–2.31)	2.84 (1.75–4.64)	2.67e-05	5.35e-05
LDL-TG Model 1^a^	Reference	1.31 (0.80–2.13)	1.39 (0.86–2.27)	3.00 (1.84–4.95)	1.38e-05	2.50e-05
LDL-TG Model 2^b^	Reference	1.43 (0.88–2.33)	1.56 (0.95–2.56)	3.36 (1.95–5.85)	1.50e-05	3.88e-05
LDL-TG Model 3^c^	Reference	1.34 (0.82–2.20)	1.48 (0.89–2.46)	3.37 (1.94–5.91)	1.86e-05	4.49e-05
LDL-TG Model 4^d^	Reference	1.44 (0.89–2.35)	1.56 (0.94–2.60)	3.42 (1.88–6.29)	6.31e-05	1.99e-04
LDL-TG Model 5^e^	Reference	1.34 (0.82–2.20)	1.46 (0.87–2.47)	3.32 (1.81–6.16)	1.19e-04	3.55e-04

CI, confidence interval; LDL-TG, low-density lipoprotein–triglycerides.

^a^Adjusted for age and gender.

^b^Adjusted for LDL-C.

^c^Adjusted for age, gender, and LDL-C.

^d^Adjusted for APOB.

^e^Adjusted for age, gender, and APOB.

### Cumulative incidence curves

**FIGURE 3:**
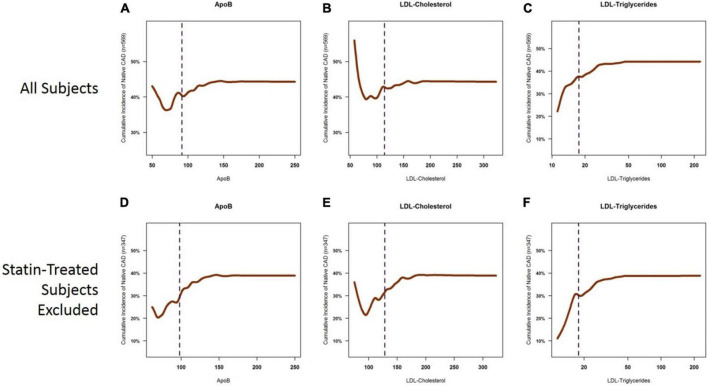
Cumulative incidence curves for the presence of coronary atherosclerosis as a function of ApoB, LDL-C, and LDL-TG. Cumulative incidence curves demonstrate the well-described relationship between ApoB and LDL-C levels and the incidence of atherosclerosis, which is primarily apparent in the ApoB range of 50–150 mg/dl and in the LDL-C range of 60–200 mg/dl. The left tails of the curves are distorted by statin-treated patients **(A,B)**, in which you see a high cumulative incidence of atherosclerosis despite very low levels of ApoB (panel A) and LDL-C **(B)**. This likely represents patients with known ASCAD whose LDL-C and ApoB levels have been lowered by aggressive statin therapy. The sigmoid relationship for ApoB and LDL-C is more apparent when statin-treated patients are excluded **(D,E)**. On the other hand, a clear, near-exponential relationship is seen for the incidence of atherosclerosis as a function of serum LDL-TG levels, with no apparent effect of statin therapy **(C,F)**. ApoB, apolipoprotein B; LDL, low density lipoprotein.

To examine the relative contribution of each key biomarker to atherosclerosis, we constructed cumulative incidence curves for ApoB, LDL-C, and LDL-TG levels against the cumulative incidence of atherosclerosis in all patients and in patients who were not on statins (Figure 3). Our data confirmed the well-described relationship between LDL-C levels and the incidence of atherosclerosis, which was most apparent in patients who were not on statin therapy (Figure 3E). A similar pattern was also observed for ApoB (Figure 3D). It is acknowledged that the left tails of the cumulative incidence curves are likely to be highly influenced by patients with known ASCAD whose LDL-C and ApoB levels were likely lowered by recent statin therapy. Importantly, statin therapy appears to have little influence on the cumulative incidence of ASCAD as a function of LDL-TG measurements (Figures 3C,F).

### Hepatic lipase (LIPC), low density lipoprotein triglycerides and atherosclerosis

**FIGURE 4:**
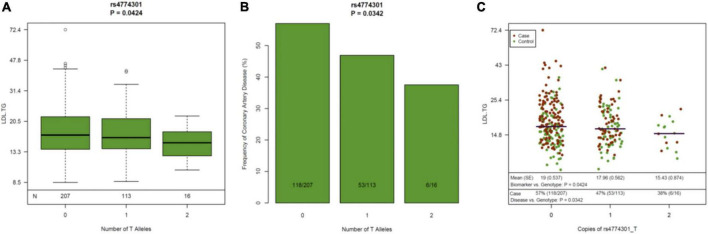
Genetic variants in the LIPC Gene, Circulating Levels of LDL-TG and Atherosclerosis. The genetic variant rs4774301 in the LIPC genomic region is associated with significantly lower circulating levels of LDL-TG **(A)** and simultaneously, with significantly lower prevalence of atherosclerosis **(B)**. Jitter plot **(C)** demonstrates the same concept in a single graph, demonstrating that the number of the “T” alleles at rs4774301 is associated with lower circulating levels of LDL-TG and with lower prevalence of ASCAD. LIPC: hepatic lipase gene; LDL-TG: low density lipoprotein triglycerides.

Since previous publications (19–21) have *demonstrated an association* between hepatic lipase (encoded by the LIPC gene), LDL-TG and atherosclerosis, we performed a genomic screen of the LIPC gene region. The SNP rs261336 was associated with both higher levels of LDL-TG and higher odds of atherosclerosis, while rs12898984, rs12900448, rs4774301, rs4775064 and rs4775065 were associated with lower circulating levels of LDL-TG and lower odds of atherosclerosis (Table 2 and Figure 4).

**TABLE 2:** Genetic association between LIPC gene variants, LDL-TG and atherosclerosis.

Variant (SNP)	LDL-TG				ASCAD (“Case”)			
		
	Beta	SE	CI	*P*-value	OR	Ln(SE)	CI	*P*-value
rs261336	0.07	0.0297	0.01_0.13	0.0223	1.5	0.2011	1.01_2.23	0.0434
rs12898984	–0.06	0.0285	–0.11 to 0.00	0.0424	0.67	0.1899	0.46_0.97	0.0342
rs12900448	–0.06	0.0285	–0.11_–0.00	0.0424	0.67	0.1899	0.46_0.97	0.0342
rs4774301	–0.06	0.0285	–0.11_–0.00	0.0424	0.67	0.1899	0.46_0.97	0.0342
rs4775064	–0.06	0.0285	–0.12_–0.00	0.0386	0.67	0.1899	0.46_0.98	0.0374

SNP, single nucleotide polymorphism. LDL-TG, low density lipoprotein triglycerides; ASCAD, atherosclerotic coronary artery disease; SE, standard error; CI, confidence interval; Ln, natural log.

**FIGURE 5:**
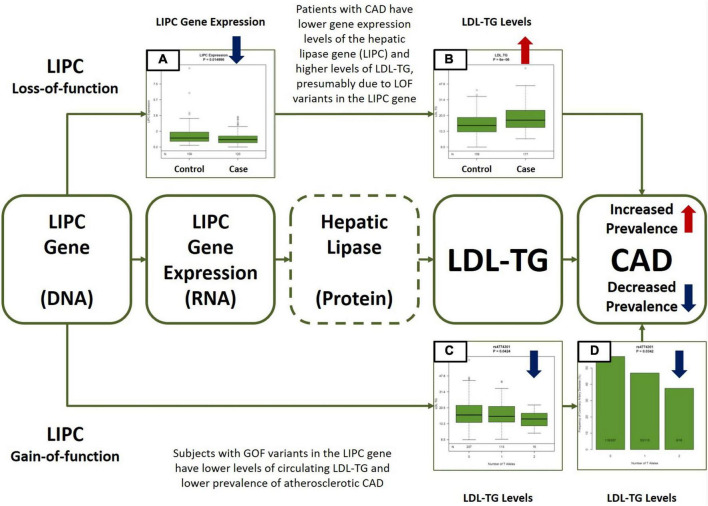
Central Illustration. Multiomic Validation of the Hepatic Lipase (LIPC)/LDL-TG Axis in Atherosclerotic CAD along the Central Dogma of Biology. Gene expression levels of hepatic lipase (LIPC) are significantly lower **(A)** and circulating LDL-TG levels are significantly higher **(B)** in patients with atherosclerotic CAD, presumably due to LOF variants in the LIPC gene (upper part of the Figure). On the other hand, GOF variants in the LIPC gene are simultaneously associated with lower circulating LDL-TG **(C)** levels AND with lower prevalence of atherosclerotic CAD **(D)**. Except for hepatic lipase activity measurements (indicated by dotted line around hepatic lipase in the Figure), the GLOBAL study database contains all other multiomic measurements along the hepatic lipase/LDL-TG/atherosclerosis axis. The overall data presented here is consistent with our hypothesis that circulating triglyceride-rich LDL particles may have a potential causal role in atherosclerosis, due to abnormal hepatic lipase activity. LIPC, hepatic lipase; LDL-TG, low density lipoprotein triglycerides; CAD, coronary artery disease; LOF, loss-of-function; GOF, gain-of-function.

In addition, LIPC gene expression in circulating mononuclear cells was significantly lower in Cases than in Controls (mean expression: 1.20 (0.08) vs. 1.49 (0.07); *p* = 0.015).

### Associations of low density lipoprotein triglycerides with other known risk markers

We analyzed lipid panel test results and lipoprotein subclass profile, determined by proton nuclear magnetic resonance (^1^H-NMR) spectroscopy, in 800 patients from the National Institutes of Health CT cohort, subdivided by LDL-TG terciles (Supplementary Table 3). Total Cholesterol and LDL-C increased from low to high LDL-TG terciles (*p* < 0.0001 for trend). The same trend was observed for triglycerides and calculated VLDL-C (18). Small dense LDL (sd-LDL) cholesterol as measured by Denka assay increased along the LDL-TG tertials. Lipoprotein subclass analysis by ^1^H-NMR spectroscopy revealed that total, large, medium, and very small triglyceride-rich lipoprotein (TRL) particle number increased from low to high LDL-TG terciles (*p* < 0.0001 for trend). Furthermore, the number of LDL particles was higher in high LDL-TG terciles at the expense of smaller LDL particles (*p* < 0.0001), probably due to the poorer sdLDL recognition by LDL receptor (22), leading to its accumulation in the plasma.

Finally, LDL-TG was positively associated with GlycA (*p* < 0.0001 for trend), a recently identified systemic inflammation marker derived from the ^1^H-NMR signal of N-acetyl groups on the glycan portion of acute-phase proteins in plasma (23). Overall, these results suggest that increased LDL-TG is linked to a more pro-inflammatory and pro-atherogenic phenotype and are, therefore, aligned with the findings from the Bayesian Network Analysis.

## Discussion

Our results suggest that, while several serum biomarkers are associated with human ASCAD, triglyceride-rich LDL particles, as measured by LDL-TG levels, may have *an important central* role, potentially as a result of abnormal hepatic lipase function. Our study design provided a unique opportunity to assess biomarker associations in the context of the impact of genetic predisposition on atherosclerosis. We completed precise and detailed quantitative phenotyping measurements of human coronary arterial atherosclerosis in a prospective study using comprehensive cardiac CT, analyzed in a central core laboratory. This precision phenotyping was coupled with measuring and ranking 99 circulating biomarkers and 37,000 “omics” measurements. We built hypothesis-free, causal Bayesian networks of biological pathways to examine the *potential role* of serum biomarkers in a comprehensive manner.

Our initial analysis identified four main biomarker clusters, thus providing unique high-level insights into the pathogenesis of ASCAD (Figure 1). The content of these clusters is consistent with prevailing hypotheses of the development of atherosclerosis as a result of atherogenic lipoproteins, inflammation, and endothelial dysfunction, in some instances in the context of insulin resistance and diabetes (24–26).

Furthermore, in univariate (Figure 1) and multivariable analyses (Figure 2A), we also found that ApoB-containing lipoproteins, insulin resistance, endothelial dysfunction, inflammation, and fibrosis are all strongly associated with human coronary atherosclerosis, consistent with decades of hypothesis-driven data.

A key finding was that, out of tens of thousands of blood-based molecules and biomarkers, LDL-TG emerged with a potential central role in human coronary atherosclerosis, potentially as a function of abnormal hepatic lipase activity. This was seen in our hypothesis-free, causal, Bayesian network analysis, which included 24,929 variables and 110,350 significant edges in the models. LDL-TG was directly connected to human coronary atherosclerosis in 95% of the models in the ensemble. The output of the Bayesian network analysis shown in Figure 2B (not an illustration) indicates the potential central role of triglyceride-rich LDL particles, as measured by LDL-TG levels. In this model, triglyceride levels and palmitoleic acid were upstream from LDL-TG, while small, dense LDL (sd-LDL) inflammatory markers (e.g., C-reactive protein, fibrinogen, and lipoprotein-associated phospholipase A2), and fibrosis markers (e.g., galectin-3) were downstream. In addition to these potentially “positive” controls, the absence of HDL-C, ApoAI, CETP and vitamin-D may serve as relevant “negative controls” in the Bayesian networks.

The hierarchal organization of the lipid/lipoprotein-related biomarkers, inflammatory biomarkers and fibrosis-related biomarkers in the Bayesian networks are consistent with a mechanistic hypothesis in which triglyceride-rich LDL particles drive downstream inflammation and a fibrotic response, directly contributing to the initiation and progression of human coronary atherosclerotic plaques (Figure 2B). It is important to emphasize, however, that our findings do not suggest that triglyceride-rich LDL particles themselves physically localize in the coronary vessel wall to initiate the atherosclerosis process. The overall lipoprotein milieu in patients with elevated LDL-TG may lead to the increased formation of sd-LDL particles, which then may physically localize to the arterial wall.

Our panomic dataset offers a unique and unprecedented opportunity to assess causality of certain biomarkers, as we can assess *simultaneous* associations between genotypes, gene expression levels, circulating biomarkers and the atherosclerotic phenotype via comprehensive cardiovascular CT. Overall, our data is consistent with the potential *central* hypothesis that loss-of-function variants in the hepatic lipase gene may be associated with lower hepatic lipase activity, higher LDL-TG levels resulting in atherosclerosis. In our data, LIPC gene expression levels were significantly lower and LDL-TG levels were significantly higher in patients with atherosclerosis. Furthermore, single nucleotide polymorphisms (SNP’s) in the LIPC gene that were associated with elevated LDL-TG levels were simultaneously associated with increased prevalence of atherosclerosis, suggesting the *potential role* of LDL-TG based on the principles of natural randomization (Figure 4) (27). Our data is consistent with historical findings that polymorphisms in the LIPC gene are associated with circulating levels of LDL-TG (28–30).

From a mechanistic point of view, our results are consistent with a potential hypothesis whereby lower hepatic lipase activity may result in decreased lipolysis, decreased remodeling, and decreased initial clearance of TRL’s, such as very low-density lipoprotein (VLDL) particles. The increased residence time of TRL’s may lead to prolonged exposure of TRL’s to CETP activity, resulting in more TG-rich IDL and LDL particles, as reflected in elevated LDL-TG levels. The presence of TG-rich IDL and LDL particles favor the generation of sd-LDL particles, which physically may localize to the arterial wall, resulting in the retention of these atherogenic lipoprotein particles, triggering an inflammatory reaction and endothelial dysfunction, culminating in the initiation and propagation of atherosclerosis.

In general, our findings are consistent with the literature but also add to those findings by demonstrating a possible *central* role of LDL-TG, potentially as a function of abnormal hepatic lipase activity, as revealed by our unique causal Bayesian network analysis and through genetic validation. A large epidemiologic study has examined the association of LDL-TG with angiographic ASCAD (31). In that study, LDL-TG levels were measured by the ultracentrifugation-precipitation method (“beta-quantification”), a cumbersome reference procedure not used for routine diagnostic testing. The main finding was that LDL-TG was a stronger predictor of ASCAD compared to LDL-C and was independent of LDL-C, with an overall odds ratio of 1.3 (95% CI, 1.19–1.43; *P* < 0.001). Although consistent with our findings, the odds ratio in our study was much higher at 3.41 (95% CI, 1.94–6.01), likely due to the use of precision phenotyping in our approach. Also consistent with our findings, they also identified significant correlations between LDL-TG and IL-6 and between LDL-TG and C-reactive protein. In a smaller more mechanistic sub-study of 114 patients, it has also been reported that in patients with high LDL-TG levels, LDL particles are enriched in triglycerides and depleted in cholesterol esters. VLDL particles showed the opposite trend; they were enriched in cholesterol esters and depleted in triglycerides. These observations are in line with the association of LDL-TG with very small TRL particle number that we observed. It is also consistent with our mechanistic hypothesis on the central role of the remodeling of apoB-containing lipoprotein particles in the development of atherosclerosis.

Several other large clinical trials have also provided important information related to the association of LDL-TG with atherosclerosis. Albers et al. examined the potential role of LDL-TG, sd-LDL and HDL subclasses in 3,094 subjects in the AIM-HIGH clinical trial (19), which was evaluating the effect of extended-release niacin in a secondary prevention population on statin background. The primary endpoint was the composite of death from coronary artery disease, non-fatal myocardial infarction, ischemic stroke, hospitalization for acute coronary syndrome or symptom-driven coronary or cerebrovascular revascularization. In their study, sd-LDL and LDL-TG were not event related. The advantage of our study is a very clear phenotype of coronary atherosclerosis based on comprehensive cardiovascular CT. In addition, the AIM-HIGH study was a secondary prevention population on the background of statin therapy, different from our patient population.

Saeed et al. also examined the potential role of LDL-TG in 9,334 subjects without prevalent CAD the ARIC study (20), using a direct homogenous assay that can be routinely applied in clinical laboratories. They found that LDL-TG were significantly associated with cardiovascular disease, even after adjusting for traditional risk factors, including lipids. This is consistent with our own findings in a similar patient population. Similarly, the authors also found that variants in the promoter region of the LIPC gene were associated with lower hepatic lipase activity, consistent with our own findings.

Finally, Silbernagel et al. (21) demonstrated that LDL-TG was associated with cardiovascular mortality in 3,140 subjects. Genome-wide association study in this cohort demonstrated that variants in the LIPC gene were significantly associated with circulating LDL-TG levels, consistent with our own findings. Furthermore, in a two-sample Mendelian randomization analysis, the authors found that low hepatic lipase activity may be the causal factor behind elevated LDL-TG levels, driving atherosclerotic cardiovascular risk. The authors suggested that LDL-TG may be on the causal pathway related to cardiovascular disease. Our combined unbiased, causal Bayesian network analysis and genomic analysis *is consistent with* these findings and *propose* a more detailed biological network explaining the hepatic lipase/LDL-TG axis of atherosclerosis (Figure 5).

In summary, we performed an unbiased, causal Bayesian network analysis to identify potential novel causal factors in human coronary atherosclerosis, revealing the potential key role of TG-rich lipoprotein particles. We then used our panomic data, including genetic validation, to further explore the potential *central* role of LDL-TG, demonstrating that the hepatic lipase/LDL-TG axis may be *an important* pathway in ASCAD.

## Limitations

Although this was a prospective, multicenter study with central core laboratory analysis of all imaging and biochemical measurements, it has some limitations. First, we only included Caucasian subjects in our study, as it was powered for genome-wide association analyses based on a single ethnic background, requiring at least 6,700 subjects (7). Second, we have limited longitudinal follow-up of the patients. Nevertheless, a key feature of Bayesian network analysis with the implementation of REFS is its ability to generate causal biological models, even in the absence of longitudinal outcomes. In addition, since we had whole genome sequence data, we were also able to demonstrate causality through genetic methods. Third, although the genetic analysis is consistent with a potential central role of the hepatic lipase/LDL-TG axis, we did not have functional measurements of hepatic lipase. Finally, although we had in *a priori* Discovery and Validation dataset in our own GLOBAL clinical study, we did not validate our findings in external datasets, technically limiting our findings to the GLOBAL clinical study population.

## Summary and conclusion

While ApoB-containing lipoproteins, inflammatory biomarkers, and markers of endothelial dysfunction and fibrosis were all associated with human coronary atherosclerosis, triglyceride-rich LDL particles, as measured by LDL-TG levels, emerged as a potentially key factor, within a sub-network that includes apoB and LDL-C. Furthermore, genetic analysis revealed the potential *central* role of the hepatic lipase/LDL-TG axis in atherosclerosis. With the recent introduction of a simple and fully automated method for the quantification of LDL-TG levels (32), this biomarker may become an important tool in the clinical assessment of patients at risk for, or with, atherosclerosis. Furthermore, the results from this study have *confirmed* a possible role of hepatic lipase in human coronary atherosclerosis, which in the future can be explored as a target for drug development. It is also already known that several approved lipid-lowering drugs, such as fibrates, and statins, have a differential effect on LDL-TG versus LDL-C (33), which will be useful to further investigate to better understand their overall impact in cardiovascular event reduction.

## Data availability statement

The Global Genomics Group, LLC company has spent in excess of $30M USD to collect the raw data that was used in the present analysis. Value creation in the company occurs by commercializing insights from the raw data, and that is how invested capital is returned to investors. Public disclosure of the raw data would compromise the full potential of value creation by the company at the present stage. Once the company returns invested capital to its investors, the raw data may be deposited in public data sources. In addition, under appropriate non-disclosure agreements, the company may disclose raw data to interested parties. Enquirers regarding raw data access are to be directed to BB, bbrown@g3therapeutics.com.

## Ethics statement

The studies involving human participants were reviewed and approved by Western Institutional Review Board, Inc. The patients/participants provided their written informed consent to participate in this study.

## Author contributions

SV, AB, DL, AR, and RN: study design, data analysis and manuscript preparation. MB: study design and statistical analysis. IM and BB: study execution. JN, PM-H, GV, IV, WH, VV, TD, DN, and BH: data analysis and manuscript preparation. AG, LF, BC, KR, and IK: data analysis. All authors contributed to the article and approved the submitted version.
