# Supplementary material for: Bayesian network analysis of panomic biological big data identifies the importance of triglyceride-rich LDL in atherosclerosis development
Source: Front Cardiovasc Med. 2023 Jan 4;9:960419. doi: 10.3389/fcvm.2022.960419 (PMC9845579; doi:10.3389/fcvm.2022.960419)
Supplement: Supplementary file 1 [file Data_Sheet_1.docx]

**Supplementary Tables**

**Supplementary Table 1. General Demographic Features.**

|  | Patients without atherosclerosis (controls;  n=317) | Patients with atherosclerosis (cases;  n=348) | *P* value |
| --- | --- | --- | --- |
| Age (yr), mean (SE) | 54.2 (0.57) | 57.6 (0.59) | <0.001 |
| Male, n (%) | 141 (44.5) | 171 (49.1) | NS |
| Typical angina n (%) | 203 (64.0) | 216 (62.0) | NS |
| Angina equivalent n (%) | 114 (36.0) | 127 (36.5) | NS |
| Diamond-Forrester Score, mean (SE) | 23.0 (1.6) | 28.5 (1.7) | NS |
| LDL-C, mg/dL, mean (SE) | 115.7 (2.0) | 111.7 (1.9) | NS |
| ApoB, mg/dL, mean (SE) | 91.3 (1.4) | 91.2 (1.4) | NS |
| HDL-C, mg/dL, mean (SE) | 56.9 (0.9) | 58.9 (1.0) | NS |
| Triglycerides, mg/dL, mean (SE) | 150.1 (5.2) | 176.5 (8.1) | NS |
| LDL-TG, mg/dL, mean (SE) | 17.2 (0.4) | 19.5 (0.7) | <0.001 |
| Hypertension, n (%) | 162 (51.3) | 249 (72.0) | <0.001 |
| Type 2 diabetes, n (%) | 23 (7.3) | 60 (17.2) | <0.001 |
| Statin use, n (%) | 105 (33.1) | 200 (57.5) | <0.001 |
| Niacin use, n (%) | 5 (1.6) | 5 (1.4) | NS |
| Fibrate use, n (%) | 18 (5.2) | 12 (3.8) | NS |
| Ezetimibe use, n (%) | 6 (1.9) | 18 (5.2) | NS |
| Fish oil use, n (%) | 27 (8.5) | 51 (14.7) | NS |
| Bile acid sequestrant use, n (%) | 3 (1.0) | 1 (0.3) | NS |

ApoB, apolipoprotein B; LDL-C, low-density lipoprotein cholesterol; LDL-TG, low-density lipoprotein‒triglycerides; NS, not significant; SE, standard error.

**Supplementary Table 2. List of Conventional Biomarkers Measured.**

| **Abbreviation** | **Full Name** | **Units** | **Manufacturer/ Method** |
| --- | --- | --- | --- |
| AA2 | Arachidonic acid | % | HDL Gas Chromatography |
| AAEPA | Arachidonic divided by EPA | % | HDL Gas Chromatography |
| ADMA | Asymmetric dimethylarginine | ng/mL | HDL Mass Spec. |
| ADMA.Arg.Ratio | Asymmetric dimethylarginine/ arginine ratio |  | HDL Mass Spec. |
| ADN | Adiponectin | µg/mL | MedTest |
| ALB | Albumin | g/dL | Beckman Coulter |
| ALP | Alkalaine phosphatase | U/L | Beckman Coulter |
| alphalin2 | Alphalinoleic acid | % | HDL Gas Chromatography |
| ALT | Alanine transaminase | U/L | Beckman Coulter |
| APO.A1 | Apolipoprotein A-I | mg/dL | Beckman Coulter |
| APO.B | Apolipoprotein B | mg/dL | Beckman Coulter |
| ApoB48 | APO Protein B48 | ng/mL | Shibayagi |
| arachidic2 | Arachidic acid | % | HDL Gas Chromatography |
| AST | Aspartate transaminase | U/L | Beckman Coulter |
| B.Sitosterol | B-Sitosterol | µg/ml | In-huse Mass Spec. |
| behenic2 | Behenic acid | % | HDL Gas Chromatography |
| C.PEP | C-Peptide | ng/mL | Roche |
| CALA | Calcium | mg/dL | Beckman Coulter |
| Campesterol | Campesterol | µg/ml | In-huse Mass Spec. |
| CHOL | Total Cholesterol | mg/dL | Beckman Coulter |
| Cholestanol | Cholestanol | µg/ml | In-huse Mass Spec. |
| cismontotl | Palmitoleic + oleic + eicosenoic + nervonic | % | HDL Gas Chromatography |
| CK8 | Cytokeratin-8 | ng/mL | HDL ELISA |
| CRE | Creatinine | mg/dL | Beckman Coulter |
| DBILC | Direct Bilrubin | mg/dL | Beckman Coulter |
| dcopentn32 | Docosapentaenoic-n3 acid | % | HDL Gas Chromatography |
| dcopentn62 | Docosapentaenoic-n6 acid | % | HDL Gas Chromatography |
| Desmosterol | Desmosterol | µg/ml | In-huse Mass Spec. |
| DHA2 | Docosahexaenoic acid | % | HDL Gas Chromatography |
| dihomolin2 | Dihomolinoleic acid | % | HDL Gas Chromatography |
| docosat2 | Docosatetraenoic acid | % | HDL Gas Chromatography |
| eicosad2 | Eicosadienoic acid | % | HDL Gas Chromatography |
| eicosen2 | Eicosenoic acid | % | HDL Gas Chromatography |
| EPA2 | Eicosapentaenoic acid | % | HDL Gas Chromatography |
| Fibrinogen | Fibrinogen | mg/dL | Kamiya |
| Folate | Serum Folate | ng/mL | Roche |
| Galectin3 | Galectin 3 | ng/mL | BG Medicine |
| gammalin2 | Gamma linoleic acid | % | HDL Gas Chromatography |
| GGT | Gamma-glutamyl transferase | U/L | Beckman Coulter |
| GLU | Glucose | mg/dL | Beckman Coulter |
| GPF | Gamma Prime Fibrinogen | mg/dL | Gamma Therapeutic |
| HbA1C | Glycated hemoglobin A1C | % | Trinity Biotech. |
| HCY | Homocysteine | µmol/L | Diazyme |
| HDL.APOE | APO E Mass on HDL | mg/dL | Denka |
| HDL.C | High Density lipoprotein Cholesterol | mg/dL | Randox (Denka reagent) |
| HDL3.C | High Density Lipoprotein Fraction 3 Cholesterol | mg/dL | Randox (Denka reagent) |
| hs.cTnI | High Sensitivity Cardiac Tropin-I | pg/mL | Quanterix |
| hsCRP | High Sensitive C Reactive Protein | mg/L | Beckman Coulter |
| IL17A.Ran | Interleukin-17A | pg/mL | Randox |
| IL17A.Sim | Interleukin-17A | pg/mL | Simoa |
| IL6 | Interleukin-6 | pg/mL | Randox |
| Insulin | Insulin | μU/mL | Roche |
| LDH | Lactate dehydrogenase | U/L | Beckman Coulter |
| LDL.TG | LDL Triglycerides | mg/dL | Denka |
| LDL.C | Low Density Lipoprotein Cholesterol | mg/dL | Beckman Coulter |
| Leptin | Leptin | ng/mL | Mercodia |
| ligno2 | Lignoceric acid | % | HDL Gas Chromatography |
| linoleic2 | Linoleic acid | % | HDL Gas Chromatography |
| Lpa.ApoA.Size | Size of ApoA in Lp(a) |  |  |
| Lpa.ApoB.percent | ApoB in Lp(a) | % |  |
| Lpa.C.Elect | Lipoprotein (a) cholesterol | mg/dL | Electrophoresis |
| Lpa.Mass | Lipoprotein (a) Mass | mg/dL | Randox (reagent made by Denka) |
| Lpa.P.Elect | Lipoprotein (a) Particle by Electrophoresis | nmol/L | Electrophoresis |
| Lpa.P.ELISA | Lipoprotein (a) Particle by ELISA | nmol/L | Sun Diagnostics |
| LpPLA2.ACT | Lipoprotein Associated Phospolipase A2 Activity | U/L | Diazyme |
| LpPLA2.Mass.DDX | Lipoprotein Associated Phospolipase A2 Mass | ng/mL | DiaDexus |
| LpPLA2.Mass.ITA | Lipoprotein Associated Phospolipase A2 Mass | ng/mL | Denka immuno-turbidmetric assay |
| MMA | Methylmalonic acid | µmol/L | HDL Mass Spec. |
| MPO | Myeloperoxidase | pmol/L | Cleveland Heart Lab |
| myristic2 | Myristic acid | % | HDL Gas Chromatography |
| NEFA | Non-esterified fatty acids | mmol/L | Wako |
| nervonic2 | Nervonic acid | % | HDL Gas Chromatography |
| NTProBNP | N-terminal pro-brain natriuretic peptide | pg/mL | Roche |
| O3Index | Omega 3 index | % | HDL Gas Chromatography |
| O3total | Omega 3 total | % | HDL Gas Chromatography |
| O6O3 | O6 total divided by O3 total | % | HDL Gas Chromatography |
| O6total | Omega 6 total | % | HDL Gas Chromatography |
| OCN | Osteocalcin | ng/mL | Roche |
| oleic2 | Oleic acid | % | HDL Gas Chromatography |
| OxLDL | Oxidized LDL (mass) | U/mL | Corgenix |
| palmitic2 | Palmitic acid | % | HDL Gas Chromatography |
| palmleic2 | Palmitoleic acid | % | HDL Gas Chromatography |
| PCSK9 | Proprotein convertase subtilisin/kexin type 9 | ng/mL | RnD system |
| PHOS | Phosphorous | mg/dL | Beckman Coulter |
| ProInsulin | ProInsulin | pmol/L | Mercodia |
| PTH | Parathyroid hormone | pg/mL | Roche |
| RBP4 | Retinol Binding Protein 4 | mg/L | Diazyme |
| RLP | Remnant Lipoproteins Cholesterol | mg/dL | Denka |
| sattotal | Total saturated fatty acids | % | HDL Gas Chromatography |
| sdLDL.C.AU5840 | Small Dense Low Density Lipoprotein Cholesterol | mg/dL | Denka, AU5840 analyzer |
| sdLDL.C.P.Mod | Small Dense Low Density Lipoprotein Cholesterol | mg/dL | Denka, Roche P Modular analyzer |
| SDMA | Symmetric dimethylarginine | ng/mL | HDL Mass Spec. |
| stearic2 | Stearic acid | % | HDL Gas Chromatography |
| TBILC | Total Bilirubin | mg/dL | Beckman Coulter |
| TNFA.Ran | Tumor Necrosis Factor Alpha | pg/mL | Randox |
| TNFA.Sim | Tumor Necrosis Factor Alpha | pg/mL | Simoa |
| tomegaprct | Total omega percent | % | HDL Gas Chromatography |
| TP | Total Protein | g/dL | Beckman Coulter |
| translin2 | Translinoleic acid | % | HDL Gas Chromatography |
| transol2 | Transoleic acid | % | HDL Gas Chromatography |
| transpalm2 | Transpalmitoleic acid | % | HDL Gas Chromatography |
| transtotal | Trans palmitoleic + trans oleic + trans linoleic | % | HDL Gas Chromatography |
| TRIG | Triglycerides | mg/dL | Beckman Coulter |
| UA | Uric Acid | mg/dL | Beckman Coulter |
| VEGF | Vascular Endothelial Growth Factor | pg/mL | Randox |
| VIT.B12 | Vitamin B12 | pg/mL | Roche |
| VIT.D | Vitamin D | ng/mL | Diasorin |

**Supplementary Table 3. Lipid panel, lipoprotein sub-fraction profile, and inflammatory markers in the NIH CT cohort divided by terciles of LDL triglycerides (LDL-TG) determined by Denka assay.**

| **Parameter** | **Low LDL-TG** | **Mid LDL-TG** | **High LDL-TG** | **P value for trend** |
| --- | --- | --- | --- | --- |
| **Total cholesterol (mg/dl)** | 163.78 (2.36) | 177.36 (2.28) | 188.97 (2.28) | *<0.0001* |
| **LDL-C (mg/dl)** | 90.74 (2.02) | 107.02 (1.94) | 121.14 (1.95) | *<0.0001* |
| **LDL-C, *Sampson* (mg/dl)** | 85.76 (2.01) | 99.34 (1.94) | 108.00 (1.94) | *<0.0001* |
| **HDL-C (mg/dl)** | 55.97 (1.02) | 54.68 (0.98) | 53.28 (0.98) | *0.1616* |
| **Triglycerides (mg/dl)** | 100.32 (7.75) | 123.82 (7.48) | 176.27 (7.48) | *<0.0001* |
| **VLDL-C, *Sampson* (mg/dl)** | 15.67 (0.86) | 20.21 (0.83) | 28.19 (0.83) | *<0.0001* |
| **sdLDL-C, *Denka* (mg/dl)** | 24.43 (0.92) | 30.80 (0.88) | 40.51 (0.89) | *<0.0001* |
| **TRL-P (nmol/L)** (24 – 240 nm) | 102.44 (5.22) | 122.96 (5.02) | 154.00 (4.98) | *<0.0001* |
| Very large TRL-P (90 – 240 nm) | 0.25 (0.05) | 0.25 (0.04) | 0.38 (0.04) | *0.0660* |
| Large TRL-P (50 – 89 nm) | 2.21 (0.43) | 3.10 (0.41) | 5.67 (0.41) | *<0.0001* |
| Medium TRL-P (37 – 49 nm) | 20.28 (1.75) | 24.24 (1.69) | 33.14 (1.67) | *<0.0001* |
| Small TRL-P (30 – 36 nm) | 33.40 (1.93) | 33.24 (1.86) | 28.33 (1.84) | *0.0917* |
| Very small TRL-P (24 – 29 nm) | 46.27 (3.85) | 62.13 (3.71) | 86.46 (3.68) | *<0.0001* |
| **LDL-P (nmol/L)** (19 – 23 nm) | 1243.25 (27.20) | 1496.54 (26.18) | 1741.31 (25.98) | *<0.0001* |
| Large LDL-P (21.5 – 23 nm) | 144.21 (10.85) | 138.86 (10.44) | 144.82 (10.37) | *0.9068* |
| Medium LDL-P (20.5 – 21.4 nm) | 552.28 (25.77) | 575.47 (24.81) | 504.99 (24.62) | *0.1217* |
| Small LDL-P (19 – 20.4 nm) | 546.79 (33.01) | 782.20 (31.78) | 1087.81 (31.54) | *<0.0001* |
| **HDL-P (µmol/L)** (7.5 – 13 nm) | 20.34 (0.22) | 20.27 (0.21) | 19.93 (0.21) | *0.3476* |
| Large HDL-P (H5+H6+H7) | 2.58 (0.12) | 2.10 (0.12) | 1.89 (0.11) | *0.0002* |
| Medium HDL-P (H3+H4) | 3.52 (0.13) | 3.56 (0.13) | 3.38 (0.13) | *0.6070* |
| Small HDL-P (H1+H2) | 14.25 (0.25) | 14.61 (0.24) | 14.66 (0.24) | *0.4408* |
| **GlycA (µmol/L)** | 372.53 (4.54) | 386.14 (4.37) | 403.05 (4.34) | *<0.0001* |
| **hs-CRP (mg/L)** | 2.44 (0.30) | 3.04 (0.29) | 3.55 (0.29) | *0.0278* |

LDL-C: low-density lipoprotein cholesterol; HDL-C: high-density lipoprotein cholesterol; VLDL-C: very low-density lipoprotein cholesterol calculated with Sampson M, et al. formula (1); sdLDL-C: small dense LDL-C determined by Denka Assay; TRL-P: triglyceride rich lipoprotein particle number; LDL-P: low-density lipoprotein particle number; HDL-P: high-density lipoprotein particle number; HDL subspecies: H7 (12.0 nm), H6 (10.8 nm), H5 (10.3 nm), H4 (9.5 nm), H3 (8.7 nm), H2 (7.8 nm), and H1 (7.4 nm); hs-CRP: high sensitivity C reactive protein.

**Reference**

1. Sampson M, Ling C, Sun Q, Harb R, Ashmaig M, Warnick R, et al. A New Equation for Calculation of Low-Density Lipoprotein Cholesterol in Patients With Normolipidemia and/or Hypertriglyceridemia. JAMA Cardiol. 2020;5(5):540-8.
